# Understanding burnout among operating room nurses: a qualitative study

**DOI:** 10.3389/fpubh.2025.1604631

**Published:** 2025-05-26

**Authors:** Yufang Li, Dan Xiang, Yun Jiang, Su Gu

**Affiliations:** ^1^Nursing Department, Anqing Municipal Hospital, Anqing, China; ^2^Yancheng Clinical Medical College of Jiangsu University/The First People's Hospital of Yancheng, Yancheng, China

**Keywords:** operating room, nurse, job burnout, influencing factors, semi-structured interview, phenomenological study

## Abstract

**Objective:**

This study aimed to explore the lived experiences of job burnout among operating room nurses and to provide an evidence-based foundation for developing targeted nursing interventions to mitigate job burnout in this population.

**Method:**

A purposive sampling method, guided by the principle of maximum variation, was employed to recruit 14 operating room nurses from a tertiary Grade A hospital in Anhui Province in January 2025. A phenomenological research approach was adopted, utilizing semi-structured interviews for data collection. Interview data were analyzed using Colaizzi’s seven-step method. Data collection continued until thematic saturation was achieved—meaning no new themes emerged in subsequent interviews.

**Results:**

Eight major themes and thirteen subthemes were extracted. Among these, five major themes were identified as factors contributing to occupational burnout among operating room nurses, while three major themes were related to coping strategies. Specifically, the findings indicated that occupational burnout among nurses was closely associated with excessive workload, insufficient emotional support, career development stagnation, and the high-pressure dynamics of healthcare relationships. Nurses commonly reported a lack of professional fulfillment and an absence of effective strategies to cope with emotional exhaustion in their work.

**Conclusion:**

Burnout among operating room nurses results from the interplay of multiple factors. This study identifies key mechanisms underlying burnout in nurses working in high-pressure environments, emphasizing the importance of improving organizational support, optimizing nurse-physician communication, and clarifying career development pathways. These findings provide a theoretical basis for the development of targeted nursing management strategies and interventions, which may effectively alleviate nurse burnout and enhance both nursing quality and team stability.

## 1 Background

In 1974, Freudenberger (1) introduced the concept of occupational burnout in psychology, defining it as a decline in an individual’s energy resulting from the overwhelming burden of others’ problems. In 1981, Maslach and Jackson (2) refined the concept, characterizing occupational burnout as a psychological syndrome defined by three core features: emotional exhaustion, depersonalization, and a diminished sense of personal accomplishment. Emotional exhaustion refers to a state of energy depletion resulting from prolonged occupational demands and is widely regarded as the central component of burnout. Depersonalization is characterized by emotional detachment and reduced empathy toward colleagues. A diminished sense of personal accomplishment reflects an individual’s decreased perception of their professional competence and the significance of their work (3). The exhaustion, cynicism, and ensuing sense of ineffectiveness experienced by individuals suffering from burnout are often collective responses to shared workplace stressors and should be viewed as a systemic issue rather than an individual failing (4). Occupational burnout represents a breakdown in the relationship between individuals and their work. It has evolved into a widespread occupational hazard and a global concern, posing a serious threat to both physical and mental health (5). Among various occupational groups, nurses are widely recognized as a high-risk population for occupational burnout. The level of burnout among nurses is significantly higher than in the general population. Moreover, its impact extends beyond the individual nurse, affecting multiple factors such as patient safety, nursing quality, patient satisfaction, nurse turnover rates, and organizational loyalty (6, 7). According to global nursing workforce surveys, the average incidence of occupational burnout among nurses was 11.23% in 2019, a figure that rose to 30.7% by 2024 (8, 9). Occupational burnout has thus become one of the key factors affecting nurses’ mental health and job performance (10).

As a specialized department within hospitals, the operating room is characterized by high work pressure, a fast-paced environment, and a closed, highly complex setting. These factors place operating room nurses under considerable psychological and physiological stress (11, 12). Multiple studies have shown that operating room nurses experience higher levels of work-related stress than nurses in other departments, and even higher levels than surgeons (13, 14). In a study by Ramuszewicz et al. (15) 32% of operating room nurses reported experiencing occupational burnout.

In China, the situation is similarly concerning. A survey conducted across eight tertiary Grade-A hospitals in Shaanxi Province revealed an occupational burnout rate of 83.16% among operating room nurses (16). Furthermore, a study by Lin Shuqiu (17) showed that severe emotional exhaustion and low personal accomplishment are common among Chinese operating room nurses, with prevalence rates of 33.6 and 47.3%, respectively. These findings indicate that operating room nurses not only face intense work-related stress, but also must cope with additional challenges related to their working environment, extended working hours, and personnel management. Despite the high prevalence of occupational burnout among operating room nurses, the issue has not received adequate attention, and relevant research remains limited (14). Most existing studies are cross-sectional, followed by intervention studies, with relatively few employing qualitative methods. Compared with cross-sectional designs, qualitative research can offer deeper insights into the experiences, perceptions, and emotions of participants, thereby providing a richer understanding of the underlying causes of burnout. Therefore, this study aims to explore the contributing factors and common coping strategies associated with occupational burnout among operating room nurses through qualitative interviews. The findings are expected to enhance the understanding of operating room nurse burnout and support the development of targeted interventions.

## 2 Study object

### 2.1 Sample selection

This study employed purposive sampling combined with the maximum variation principle to select nurses from the operating room of a tertiary first-class hospital in Anhui Province as research participants. In the selection process, factors such as age, gender, job title, education level, and years of work experience were considered to ensure the representativeness and diversity of the sample. In the study, we administered a questionnaire survey using the revised Chinese version of the MBI-GS scale by Li Chaoping (18) to the operating room nurses at the hospital. This scale has been widely used in China and has demonstrated good reliability and validity (19). According to the weighted average score classification standard developed by Kalimo et al. (20), a participant is considered to exhibit symptoms of burnout if the scores for all MBI-HGS scale items are ≥1.5. To better understand the causes of burnout among operating room nurses, we included nurses with symptoms of burnout in the study.

Inclusion criteria: Nurses who had worked in the operating room for at least 1 year; exhibited symptoms of burnout; and possessed normal cognitive function and communication abilities. Exclusion criteria: Rotating nurses and visiting nurses; nurses who were absent from work due to personal leave, sick leave, or maternity leave; and nurses who refused to participate in the study.

### 2.2 Determination of sample size

The sample size was determined based on the principle of data saturation, meaning that sampling was considered complete when no new themes or information emerged during the interviews. The degree of data saturation was the key criterion for determining the sample size. If additional participants no longer contributed new information, data were deemed to have reached saturation. A total of 14 participants were ultimately included in this study.

### 2.3 Research instruments

This study employed semi-structured interviews to collect data. The interview questions were developed based on Maslach’s three-dimensional theory of burnout-emotional exhaustion, depersonalization, and a diminished sense of personal accomplishment, along with a review of relevant literature, to ensure that the data collected effectively reflected the multi-dimensional factors of occupational burnout. After an initial group discussion to determine the preliminary interview outline, pre-interviews were conducted with two operating room nurses. Based on the results of these pre-interviews, the interview outline was appropriately modified to ensure its completeness and adaptability. The final version of the interview outline was then established (see Table 1).

**Table 1:** Interview outline.

1	Have you been feeling stressed at work recently? Can you tell me more about what’s been causing it?
2	Why did you become an operating room nurse? Can you explain it more?
3	Could you describe your current work situation in detail?
4	Have you experienced feelings of exhaustion or low mood at work?
5	Could you elaborate on that?
6	Do you find your job as an operating room nurse fulfilling?
7	What do you believe are the reasons in your work that make you feel exhausted or low in mood?
8	What do you consider to be the main factors contributing to job burnout?
9	Which of these factors do you think is the most important?
10	How has job burnout affected your life and work?
11	How do you cope when you experience such situations?

### 2.4 Data collection methods

Semi-structured interviews were employed for data collection in this study. Before the interviews, the researchers thoroughly explained the purpose, content, and confidentiality principles of the study to all participants to ensure informed consent, and written consent forms were signed. Upon obtaining consent, specific interview times were scheduled.

The interviews were conducted in the head nurse’s office, providing a quiet and comfortable environment that minimized external interruptions and ensured that the conversations could not be overheard by unrelated personnel. All interviews were audio-recorded in their entirety. The researchers maintained a neutral stance throughout, avoiding interruptions or leading influences. Careful attention was paid to the participants’ tone of voice, facial expressions, and body language, and participants were encouraged to freely express their feelings. Follow-up questions and clarifications were asked when appropriate. Each interview lasted between 30 and 45 min to ensure a deep understanding of the participants’ genuine thoughts and emotions.

### 2.5 Data analysis methods

All interview recordings were transcribed within 24 h and anonymized to ensure confidentiality. Two researchers (DX and YJ) independently conducted open coding to generate initial codes. The research team then reviewed the codes collaboratively, resolved discrepancies through discussion, and repeatedly reflected on the research questions to reach consensus. Data were analyzed using a phenomenological approach following Colaizzi’s seven-step method (21), which involved: extracting significant statements from the transcripts; coding recurrent viewpoints to identify initial themes; grouping similar statements into meaning units; reviewing and refining these units to ensure alignment with participants’ perspectives; clustering meaning units into thematic categories; conducting constant comparisons to validate consistency and reliability; and finally, summarizing the core themes related to occupational burnout and clarifying their internal relationships.

### 2.6 Ethics

This study was approved by the Medical Ethics Committee of Anqing Municipal Hospital (approval number: Medical Ethics Review [2025] No. 1). All participants provided informed consent and voluntarily participated in the study.

## 3 Results

### 3.1 General information about the interviewees

In this study, interviews were conducted with 14 participants. No new information emerged during the later stages of data collection, indicating that data saturation had been achieved; therefore, the interviews were concluded after 14 operating room nurses had been interviewed. Participants were coded sequentially according to the order of their inclusion in the study (N1–N14). Among them, 78.57% were female. Participants’ ages ranged from 24 to 45 years, with a mean age of 31.79 years. Their professional experience ranged from 2 to 15 years, with an average of 8.07 years. Notably, 50% of the participants held the title of charge nurse. Detailed demographic characteristics of the participants are presented in Table 2.

**Table 2:** General data survey form of the research subjects.

Participant ID	Gender	Age	Degree of education	Professional title	Operating room experience
N1	Female	39	Bachelor	Supervisor nurse	13
N2	Female	28	Bachelor	Junior nurse	5
N3	Female	24	Associate degree	Junior nurse	2
N4	Female	27	Bachelor	Junior nurse	4
N5	Female	32	Bachelor	Supervisor nurse	10
N6	Female	36	Bachelor	Supervisor nurse	13
N7	Female	26	Bachelor	Junior nurse	4
N8	Female	35	Bachelor	Supervisor nurse	12
N9	Female	28	Bachelor	Junior nurse	5
N10	Female	45	Bachelor	Associate senior nurse	15
N11	Female	29	Bachelor	Supervisor nurse	5
N12	Male	26	Associate degree	Junior nurse	3
N13	Male	35	Bachelor	Supervisor nurse	11
N14	Male	35	Bachelor	Supervisor nurse	11

### 3.2 Interview results

The interview data were organized and analyzed around two primary dimensions: factors contributing to occupational burnout among operating room nurses, and strategies for addressing it. Five major themes were identified for the first dimension, and three for the second. A summary of the results is provided in Table 3, with detailed thematic descriptions discussed in the following sections.

**Table 3:** Interview results.

	Category	Subcategory
Factors influencing burnout among operating room nurses	Personal factors	Personality traits and self-expectations
		Low professional identity — lack of passion and passive career choice
		Psychological Burden and Emotional Distress
		Role conflict and time management challenges
	Organizational structure and management factors	Limited opportunities for promotion and training
		Management factors
		Mismatch between effort and reward
		Unreasonable scheduling and human resource allocation
	Interpersonal Relationship Factors	High pressure in interpersonal communication
		Tension in doctor-nurse relationships and workplace oppression experiences
	Lack of family support and societal misperceptions	/
	Professional characteristics factors	High-intensity workload
		Emotional burden and psychological impact
		High repetitiveness and low autonomy
Coping strategies for job burnout	Engaging in self-adjustment	/
	Seeking social support	/
	Reflection and adjustment	/

#### 3.2.1 Factors influencing burnout among operating room nurses

##### 3.2.1.1 Theme 1: personal factors

###### 3.2.1.1.1 Personality traits and self-expectations

Individual personality characteristics and mental health conditions, such as perfectionism, anxiety, and strong self-imposed demands, made nurses more susceptible to experiencing greater stress at work. In addition, some nurses lacked effective psychological adjustment skills, showed slower emotional recovery, or exhibited insufficient intrinsic motivation and passion for their profession. These factors increased their psychological and physical stress, further leading to emotional exhaustion.

N4 stated: “I always want to do everything perfectly, so I constantly feel that what I do is never good enough.”

###### 3.2.1.1.2 Low professional identity — lack of passion and passive career choice

Some nurses did not choose the nursing profession based on their own interests but rather due to external pressures or passive academic reassignment. Their limited understanding of the profession led to a low sense of professional identity. The gap between their expectations and the reality of nursing work also became a significant source of burnout.

N2 stated: “I originally wanted to study clinical medicine, but my entrance exam score was not high enough, so I was reassigned to nursing. I have little interest in nursing; I just had to accept it.”

N3 shared: “Choosing nursing was mostly arranged by my family because they thought it was a stable profession. I imagined nursing to be like what I saw on TV—sacred and heartwarming—but the reality is endless night shifts.”

Entering the profession passively, combined with the stark contrast between the ideal and the actual work experience, made it difficult for nurses to develop intrinsic passion and a clear sense of purpose, ultimately affecting their sense of achievement.

###### 3.2.1.1.3 Psychological burden and emotional distress

Some nurses mentioned experiencing significant psychological pressure and emotional distress when dealing with emergencies, particularly when worrying about intraoperative complications or accountability for postoperative adverse events.

N8 stated: “When I get home, I always fear receiving a call from a colleague — like if there’s an intraoperative pressure injury or if they are asking whether the surgical sponges were counted properly. Even though we followed the protocol and counted them, I still feel afraid when asked.”

N1 added: “I’m most afraid of assisting in neurosurgery. The surgery takes a long time, and the patient’s position is awkward, which increases the risk of pressure ulcers.”

N8 further shared: “There are so many types of medical devices, and they are updated quickly. For example, there are over ten types of intraocular lenses in ophthalmology, and I’m especially afraid of picking the wrong one.”

In addition to these concerns, nurses also worry about occupational hazards such as infection risks, radiation exposure, and surgical smoke. They fear that the work environment may jeopardize their physical health.

N5 expressed: “I do not even know if the lead walls in the operating room are really effective. We’re in this environment every day, and I’m worried about my own health.”

N8 shared: “A few days ago, I got a needle-stick injury, and the patient had syphilis. I had to get the long-acting penicillin shot.”

###### 3.2.1.1.4 Role conflict and time management challenges

The interviewed operating room nurses generally reported that with the transformation of nursing work models and the increasing demands of their roles—particularly the strengthened requirements for clinical teaching and research capabilities—they faced significant conflicts among their professional, family, and academic responsibilities. The emphasis on research output as a key criterion for promotion further intensified their occupational burden.

Under the heavy workload, nurses often struggled to balance the demands of extended working hours, heavy research and teaching tasks, and family responsibilities, leading to increasingly prominent time management difficulties. Most nurses noted that although additional tasks outside routine duties (such as department quality control and teaching assignments) initially brought a sense of achievement, the long-term imbalance between the high level of effort and limited rewards eventually led to emotional exhaustion. Meanwhile, excessive overtime work squeezed the time available for family interactions, triggering role imbalance at home and deep feelings of guilt. The interaction of these multiple stressors became a critical factor contributing to professional burnout.

N1 commented: “The hardest part is not the regular work but completing all those additional tasks. At first, it gave me a sense of accomplishment, but over time, the imbalance between effort and reward made me feel resentful.”

N9 shared: “My daily working hours far exceed eight hours. I have little time to spend with my family or handle personal matters, and the low salary adds to my family’s dissatisfaction.”

N14 expressed: “I have no time to accompany my child or help with their studies. I feel deeply guilty and often sense that both my family life and work are out of balance, leaving me feeling defeated.”

##### 3.2.1.2 Theme 2: organizational structure and management factors

###### 3.2.1.2.1 Limited opportunities for promotion and training

Operating room nurses reported facing significant challenges in their career development, such as restricted promotion pathways, high pressure to undertake research tasks, limited training opportunities, anxiety stemming from professional title evaluation reforms, and a lack of organizational support. These factors contributed to feelings of confusion and anxiety regarding their future career prospects. Unable to change their circumstances in the short term, many nurses expressed frustration and even despair.

For example, N10 mentioned, “I feel that it is harder for nurses in the operating room to get promoted compared to those in general wards.” N7 expressed uncertainty about the future: “I do not know what I should do going forward. It feels like there is no room for advancement in nursing. Sometimes I think about resigning, but I do not know what I could do after that. I want to change the current situation, but I do not know how.” Similarly, N13 stated, “Now, professional title promotions are tied to research output, and for regular nurses like us, it remains very difficult to get promoted.” N1 also commented, “It feels like there is no future. Doctors have opportunities to pursue further training, but we hardly ever get such chances to learn.” In addition, N7 reflected, “The hospital expects us to produce research outcomes, and professional titles and promotions are closely linked to research. However, the hospital does not provide sufficient resources to support these efforts.”

These sentiments reflect a widespread sense of confusion among nurses regarding career development in the healthcare field, especially in the context of a rapidly evolving medical environment, where nurses often lack a clear understanding of their career pathways and development opportunities.

###### 3.2.1.2.2 Management factors

Unfair practices within the organization, a lack of transparency in institutional policies, absence of effective incentive mechanisms, and complex interpersonal relationships further intensified nurses’ sense of alienation. Several nurses reported that their efforts were not recognized, that promotion processes were opaque, and that favoritism from leadership was common.

N6 stated, “Sometimes I feel an uncontrollable urge to lose my temper. It feels like this job has no meaning. The head nurse is very biased, and my work is not recognized by others. This makes me very sad. Sometimes I feel frustrated, sometimes angry, and I think I might be becoming depressed.”

N14 shared, “I think I performed quite well, but during the recent team leader selection, the management chose someone with less seniority than me—nurse A. I feel she is not as capable as I am, and it really made me feel very disappointed.”

When individual efforts go long unacknowledged by the organization, it can easily lead to feelings of devaluation and alienation, further weakening nurses’ sense of belonging and loyalty to the organization.

###### 3.2.1.2.3 Mismatch between effort and reward

Operating room nurses commonly face high-intensity and long-duration workloads, but they lack corresponding support in terms of salary, career advancement, and social recognition, which creates a significant sense of psychological imbalance. This imbalance not only diminishes nurses’ enthusiasm for their work but also contributes to the loss of professional fulfillment. The respondents generally reported taking on multiple responsibilities beyond their primary nursing duties, including quality management, teaching guidance, research tasks, and administrative coordination. The constant accumulation of tasks and the expansion of role expectations increased nurses’ emotional labor, yet the institutional incentives and support resources were notably insufficient. As a result, nurses experienced a strong sense of “effort-reward imbalance.”N5 described, “I am mainly responsible for the department’s quality control work and often have to complete tasks during my rest time. When problems arise, I lack support, which often leaves me feeling helpless.”N1 pointed out, “The most exhausting part is not the daily nursing work, but the completion of various additional tasks. In the beginning, I might feel a short-lived sense of accomplishment, but as the effort and rewards become disproportionate, my emotions shift from positive to resistant. The low salary, combined with my family’s lack of understanding of my work, further exacerbates the internal pressure.”

###### 3.2.1.2.4 Unreasonable scheduling and human resource allocation

Some interviewed nurses reported that scheduling in the operating room was arbitrary, with inconsistent working hours. The irregular work arrangements disrupted nurses’ established life routines, severely affecting their ability to plan personal time and fulfill family responsibilities.

The disorganization of daily rhythms not only made it difficult for nurses to manage their personal affairs but also further intensified the conflict and tension between their professional and family roles.

N1 remarked, “The most frustrating thing is not having a fixed time to leave work, which makes it impossible for me to arrange my personal life.”

N9 stated, “My daily working hours are excessively long, far exceeding eight hours, leaving me unable to spend time with my family or handle personal matters.”

N14 shared, “I feel like I barely have any time to spend with my child, and I cannot keep up with their education. Sometimes I feel extremely guilty, as if I am failing both at work and at home.”

Unreasonable human resource allocation and poor scheduling practices have kept nurses in a constant state of time pressure and mental fatigue. The boundaries between work and personal life have become increasingly blurred, leading to continuous psychological stress. In such conditions, it becomes difficult for nurses to transition effectively between different roles, resulting in heightened emotional exhaustion and intensified professional burnout.

##### 3.2.1.3 Theme 3: Interpersonal relationship factors

###### 3.2.1.3.1 High pressure in interpersonal communication

Some interviewed nurses reported experiencing significant interpersonal communication pressures within the operating room. Misunderstandings and conflicts often arose during coordination between nurses and physicians (including surgeons and anesthesiologists), as well as among nurses themselves, negatively affecting work emotions and the overall atmosphere of team collaboration.

Tense interpersonal relationships were identified as a major emotional burden beyond the inherent stress of the job itself. N4 stated, “The hard work itself is not frightening; what is truly daunting is dealing with complex interpersonal relationships, where a slight misstep can easily offend others.”

N8 similarly remarked, “The interpersonal dynamics in the operating room are very complicated. We frequently need to communicate and coordinate between anesthesiologists, surgeons, and others. Moreover, since there are many nurses in our department, even small oversights can easily lead to dissatisfaction among colleagues.”

The high frequency and high sensitivity required in interpersonal communication demand constant emotional regulation and relationship management from operating room nurses. Prolonged exposure to such a high-alert and tense interpersonal environment not only consumes considerable emotional resources but also diminishes nurses’ positive experiences at work, becoming an important psychosocial factor contributing to professional burnout.

###### 3.2.1.3.2 Tension in doctor-nurse relationships and workplace oppression experiences

In the operating room, which is typically structured around a doctor-centered organizational model, nurses often occupy a subordinate, marginal position, lacking the necessary voice and participation in decision-making processes. The imbalance in doctor-nurse relationships directly impacts nurses’ psychological sense of safety and their professional experience.

Several interviewed nurses reported experiencing a clear sense of oppression and control in their interactions with surgeons. Some nurses even described instances of workplace disrespect, including emotional abuse and sexual harassment.

N3 shared, “I am really terrified of Dr. A’s surgeries. I have to prepare a day in advance, and even then, I worry about making mistakes. Once, I could not keep up with the pace, and he frowned and sighed. I felt like I was about to break down.”

N5 also expressed, “I really do not want to go to work recently. Just thinking about having to assist Dr. B makes me anxious. If a surgery does not go well, he takes out his anger on us. The pressure is so overwhelming that I cannot sleep at night.”

N6 added, “Doctors never listen to our suggestions. Whenever they arrange surgeries, they only communicate with anesthesiologists and never consider nurses’ opinions. To them, we are invisible.”

Additionally, some nurses reported experiencing sexual harassment, including inappropriate comments, sexually suggestive jokes, and even unsolicited physical contact. N3 admitted, “Some doctors make really inappropriate jokes that make me feel embarrassed and uncomfortable. But since they are department heads, I just have to tolerate it and cannot respond directly.”

These negative experiences have severely eroded nurses’ professional dignity and psychological sense of safety, deepening their resentment toward the work environment and their emotional detachment from their professional roles. In the high-pressure, hierarchical environment of the operating room, nurses are prone to emotional exhaustion, role alienation, and a loss of self-worth. The cumulative effect of repetitive tasks, lack of role autonomy, insufficient organizational support, and poor doctor-nurse relationships creates the psychosocial conditions for the development of depersonalization, further diminishing nurses’ professional identity and sense of belonging.

##### 3.2.1.4 Theme 4: lack of family support and societal misperceptions

Operating room nurses commonly face societal misperceptions and a lack of support from their families, which further weakens their sense of professional identity and pride. Many interviewed nurses reported that the value of the nursing profession is not fully understood or respected by family members and the general public, leading to a diminished sense of professional dignity and a decline in self-confidence.

N1 remarked, “The social status of nurses is too low. Sometimes it feels like people in society do not respect us enough, and some even show disdain, which makes me feel very inferior.”

N5 shared, “My parents never tell others that I am a nurse. They always think this job is less prestigious than being a doctor, and sometimes they even feel embarrassed about it.”

N8 also commented, “Most people still only recognize the contributions of doctors and treat nurses differently. This makes me feel especially wronged.”

This dual pressure from family and society not only erodes nurses’ professional dignity but also subtly weakens their professional confidence and sense of self-worth. The lack of external recognition, combined with a persistent imbalance between effort and reward, exacerbates the emotional detachment nurses feel toward their professional roles.

The low sense of personal achievement is particularly prominent among operating room nurses. The highly repetitive nature of the work, the lack of autonomy, and the absence of positive feedback mechanisms make it difficult for nurses to fulfill their need for self-actualization. Meanwhile, the lack of emotional support from both society and family, alongside the ongoing imbalance between effort and reward, further deepens their emotional alienation from their professional roles.

Under the combined influence of these factors, nurses gradually fall into a vicious cycle of damaged professional identity, emotional exhaustion, and deep burnout, ultimately affecting their career longevity and mental health.

##### 3.2.1.5 Theme 5: professional characteristics factors

###### 3.2.1.5.1 High-intensity workload

Some nurses indicated during interviews that working in the operating room entails immense pressure, including intensive surgical assistance (such as maintaining high levels of concentration during procedures), frequent night shifts, prolonged standing, the physical demands of moving patients and surgical equipment, and shortages of resources and supplies. These factors significantly increase the physical and mental burdens of nurses and are key contributors to emotional exhaustion.

N3 stated, “Certain surgeries, such as cardiac procedures, require extremely high standards and prolonged concentration. By the end of the day, I feel utterly drained.”

N6 mentioned, “Night shifts are too frequent, and there are many emergencies at night. I am especially anxious when facing critically ill patients—the pressure is overwhelming.”

N8 reflected, “Surgical procedures can last for hours, requiring nurses to stand for long periods and maintain a high level of physical and mental output, often leading to severe physical and psychological fatigue.”

N14 explained, “We frequently have to move and prepare surgical instruments, and reposition heavier patients or adjust them into special positions, all of which demand considerable physical effort.”

N5 added, “When there are many surgeries, supply shortages are common. I have to spend a lot of time searching for equipment, which is extremely frustrating.”

###### 3.2.1.5.2 Emotional burden and psychological impact

Some nurses reported experiencing significant emotional distress when dealing with critically ill patients, unexpected emergencies, poor surgical outcomes, or death. These emotionally charged scenarios can easily lead to emotional exhaustion.

N2 shared, “Sometimes when a surgical patient has a poor prognosis, it makes me feel deeply saddened, and it ruins my mood for the entire day.”

###### 3.2.1.5.3 High repetitiveness and low autonomy

Many participants reported that work in the operating room is highly standardized and repetitive. Tasks such as preparing instruments, maintaining sterile fields, and setting up the operating room are nearly identical each day, lacking in challenge and variety. This monotonous, mechanical work pattern gradually diminishes nurses’ sense of professional achievement and contributes to emotional fatigue. N2 noted, “My work is just endless repetition; there’s nothing new or exciting about it.”N10 also stated, “Every day we repeat the same tasks—preparing surgical instruments.”

Furthermore, nurses’ tasks are highly dependent on the lead surgeon’s directives, leaving little room for independent decision-making. Over time, they often perceive themselves as mere “tools,” executing others’ instructions.

N13 commented, “We just do whatever the doctors ask. Over time, it feels like we are machines, with no sense of accomplishment.”

This doctor-centered work model erodes nurses’ professional agency and undermines their sense of identity and value. In a working environment that lacks autonomy, creativity, and humanistic care, nurses gradually lose emotional engagement with patients, viewing them more as tasks than individuals. This leads to emotional numbing and a loss of professional passion.

Such phenomena reveal a deeper cause of burnout: the chronic psychological erosion caused by structural systems and rigid role definitions. Long-term engagement in low-autonomy, low-variability tasks gradually weakens nurses’ recognition of their professional value, further diminishing their enthusiasm and satisfaction with their careers.

#### 3.2.2 Coping strategies for job burnout

In the high-pressure, repetitive, and emotionally exhausting environment of the operating room, some nurses demonstrate proactive individual coping strategies, including self-adjustment, seeking social support, and engaging in self-reflection. By diverting their attention, participating in leisure activities, expressing emotions, and seeking understanding and assistance from family members, friends, and supervisors, nurses are able to alleviate, to some extent, the negative effects of occupational burnout. These coping mechanisms reflect the psychological resilience and self-repair capacity of individual nurses. However, due to limited external support systems and insufficient improvements in organizational environments, individual efforts often fail to address the deeper causes of burnout fundamentally. Therefore, beyond nurses’ self-regulation, institutional support and organizational-level interventions are urgently needed to effectively promote the sustainable development of nursing careers and safeguard nurses’ mental health.

##### 3.2.2.1 Theme 1: engaging in self-adjustment

Some interviewed nurses reported that when facing occupational stress, they adopted self-adjustment strategies such as self-comforting and distraction to alleviate emotional distress. N7 mentioned, “Many times, I relieve stress by decompressing myself, such as doing things I enjoy or traveling.” Similarly, N2 stated, “Sometimes I comfort myself by thinking that perhaps others are experiencing the same difficulties; everyone faces unavoidable challenges at work.” These self-adjustment strategies helped nurses to some extent in mitigating the negative emotions associated with work, though their effectiveness was often limited and largely dependent on individual psychological resilience and coping mechanisms.

##### 3.2.2.2 Theme 2: seeking social support

Some nurses indicated that when dealing with work-related stress and emotional distress, they actively sought emotional support and understanding from family members, friends, or colleagues. N14 shared, “When I feel sad, I talk to my family and seek their comfort, although sometimes they do not fully understand. I also confide in a few close friends, and afterward, I usually feel a bit better.” In addition, some nurses reported seeking support and assistance from their direct supervisors. N2 recounted, “There was a time when I told the head nurse that I no longer wanted to circulate in ophthalmic surgeries because the pressure was too great and it was causing me insomnia. After that conversation, I wasn’t assigned to ophthalmic surgeries for about 2 weeks, which gave me some relief.”

##### 3.2.2.3 Theme 3: reflection and adjustment

Some nurses stated that when encountering professional stress and emotional challenges, they engaged in self-reflection by evaluating their work status and emotional experiences, seeking ways to adjust and adapt, and exploring better approaches to achieving a balance between work and life.

N3 remarked, “I calm myself down and take time to carefully reflect on my work situation before starting again.”

Similarly, N5 shared, “Sometimes I choose to take a break, and sometimes I reflect on myself to see if there are areas where I can improve.”

## 4 Discussion

This study systematically explored the multidimensional factors influencing occupational burnout among operating room nurses through semi-structured interviews. The findings revealed that occupational burnout is not merely an external manifestation of individual psychological responses but rather the result of complex interactions among personal, organizational, and social environmental factors.

### 4.1 Work environment and organizational factors: external drivers of occupational burnout

This study found that the high-intensity, fast-paced, task-concentrated, and irregular nature of operating room work is a significant external factor contributing to occupational burnout. Most nurses reported common issues such as the lack of stable off-duty hours and frequent overtime. In particular, scrub nurses, often with less seniority, repetitive tasks, and a weaker sense of participation, experienced more pronounced feelings of occupational fatigue and a loss of achievement. These findings are consistent with those of Cavuoto et al. (22), who identified long working hours and a lack of task challenge as important predictors of occupational burnout. In addition, the irrational structure of the nursing promotion system further exacerbates feelings of professional frustration. In practice, career advancement is overly dependent on research achievements, while resource support remains limited, leaving many nurses facing a “dead-end” in their career progression. Previous studies (23, 24) have also reported a positive correlation between research-related anxiety and nurse burnout. This study further confirms that, in such a context, career development barriers become a major source of burnout, even threatening the stability of healthcare service quality.

### 4.2 Family pressure and social cognitive bias: underrecognized systemic impacts

This study further emphasizes the impact of family-related factors and social cognitive biases on occupational burnout. Nurses often carry negative emotions from the workplace into their family lives, leading to strained intimate relationships, which in turn exacerbates their psychological burden, creating a vicious cycle of work–family conflict. Issues such as childcare responsibilities, financial stress, and family members’ health problems were commonly cited by nurses as contributing factors to burnout. These findings are consistent with previous literature (25), which has also highlighted the significant influence of family pressures on occupational burnout.

At the same time, widespread societal misconceptions about the nursing profession—such as stereotypes of low status and weak professionalism—undermine nurses’ sense of respect and pride, intensifying feelings of identity loss and value depreciation. This culturally rooted pressure is particularly pronounced in China’s collectivist context and serves as an important supplement to the findings of Teymoori et al. (26), which did not fully address this dimension.

### 4.3 Interpersonal relationships and workplace culture: persistent sources of implicit stress

As a highly collaborative team environment, the atmosphere and interpersonal relationships in the operating room significantly impact nurses’ psychological well-being. During interviews, several nurses reported negative experiences during interactions with surgeons, including power imbalances, emotional criticism, and even verbal abuse, resulting in persistent psychological stress. Studies indicate that 92.1% of operating room nurses in China have experienced psychological violence, with approximately 30.9% of this abuse coming from surgeons (27). This not only undermines the nurse-physician collaboration but also significantly increases nurses’ burnout levels and turnover intentions. Additionally, workplace bullying and poor communication were frequently mentioned as contributing factors. Research has shown that effective communication skills and team collaboration mechanisms can significantly alleviate nurse burnout (28–30). This study also found that in work environments with supportive management and positive interpersonal interactions, nurses are more likely to receive emotional support, thereby reducing the risk of burnout. Therefore, hospital administrators should proactively intervene to optimize communication mechanisms, prevent workplace violence, and alleviate the psychological toll of interpersonal stress by fostering an egalitarian, respectful, and collaborative team culture.

### 4.4 Professional identity and career development: pathways to stimulating intrinsic motivation

Professional identity serves as an important psychological resource through which nurses perceive the value and meaning of their work. Research has shown that the stronger the sense of professional identity, the more likely individuals are to experience a sense of achievement, mission, and responsibility, leading to higher levels of work engagement and lower levels of burnout (31, 32). This study found that some nurses exhibited low levels of professional identity, often due to having entered the nursing profession involuntarily or experiencing a sense of value loss from long-term repetitive work. At the same time, an unclear career development pathway and limited opportunities for advancement emerged as important mechanisms contributing to burnout. Although many nurses expressed a desire to pursue growth through continuing education and professional training, insufficient organizational support and the absence of structured career planning have led to bleak career prospects, resulting in feelings of helplessness, despair, and even intentions to leave the profession. This study further analyzes the intrinsic coupling between professional identity and career development, and emphasizes the need for institutional interventions to support and improve these areas.

Drawing on existing literature, this study is the first qualitative investigation to examine the multidimensional factors contributing to occupational burnout among operating room nurses in China. Using semi-structured interviews, we conducted an in-depth analysis of the complex interplay between individual, organizational, and sociocultural factors, offering new insights into the lived experience of burnout. In contrast to prior research that often focuses on a single level of analysis, this study introduces a comprehensive, multi-level framework that captures key contributors such as family responsibilities, systemic barriers to career advancement, and cultural stigma associated with the nursing profession. This framework provides a theoretical foundation for the development of more targeted, context-specific interventions and offers practical implications for policy reform and organizational practice.

## 5 Limitations

Although this study provides valuable insights into occupational burnout among operating room nurses, several limitations should be noted.

Firstly, the sample in this study was primarily drawn from operating room nurses in a specific region, and the regional nature of the sample limits the generalizability of the findings. Future research could be conducted in different regions or types of hospitals to further validate the applicability of these findings.

Secondly, this study employed qualitative interviews, which allowed for an in-depth exploration of nurses’ subjective experiences and perceptions. However, due to the limited sample size, it did not fully capture the diversity and individual differences among all nurses. Future studies could combine quantitative methods to quantify the factors associated with burnout, thereby improving the universality and representativeness of the findings.

## 6 Conclusion

This study identified key factors driving occupational burnout among operating room nurses through qualitative interviews. Burnout was found to result from a multifaceted interplay of personal stressors, organizational challenges, and weakened professional identity. Major contributors included family pressures, repetitive and high-stress work conditions, poor communication, workplace violence, and unclear career development paths. In contrast, strong professional identity, supportive work environments, and adequate social support helped mitigate burnout and enhance motivation. These insights underscore the need for comprehensive, multidimensional interventions to improve nurse well-being, job satisfaction, and workforce retention.

## Data Availability

The original contributions presented in the study are included in the article/supplementary material, further inquiries can be directed to the corresponding author.
